# Editorial: Exploring the Frontiers of Regenerative Cardiovascular Medicine

**DOI:** 10.3389/fcvm.2019.00013

**Published:** 2019-02-26

**Authors:** Julie A. Phillippi, Elena Aikawa, Josh Hutcheson

**Affiliations:** ^1^Departments of Cardiothoracic Surgery and Bioengineering, University of Pittsburgh, Pittsburgh, PA, United States; ^2^Brigham and Women's Hospital, Harvard Medical School, Boston, MA, United States; ^3^Florida International University, Miami, FL, United States

**Keywords:** cardiovascular disease, regenerative medicine, calcific aortic valve, cardiovascular calcification, aneurysm, stem cells, extracellar matrix

In this special issue of Frontiers in Cardiovascular Medicine, we assembled a collection of original research articles (4), reviews (6), a case report and a perspective within an over-arching theme of “Exploring the Frontiers of Regenerative Cardiovascular Medicine.” This collection of work engaged multiple disciplines. From this set of articles, we identified two emerging themes representing current knowledge and active progress within research domains related to regenerative medicine in the cardiovascular space. The first theme explores recent basic science efforts in uncovering new disease mechanisms, addresses challenges of working in human pathologic tissue and cell culture models and identifies novel theories in cardiovascular pathophysiology. The second theme encompasses recent translational strides. In this issue, a perspective article from investigators of the International Society for Applied Cardiovascular Biology (ISACB) by Hutcheson et al. details significant progress in applying cardiovascular biology in translational directions. A comprehensive retrospective first takes us through the work of international experts since the first human heart transplant in Cape Town, South Africa. This benchmark event in 1967 was commemorated 20 years later when this society was born and then 50 years later in 2017 when scientists reconvened in Cape Town for a celebration and international convention on cardiovascular disease. Motivated by the topics discussed at this commemorative assembly, this comprehensive perspective takes us through the past 50 years, highlighting the pioneering and innovative efforts of the scientific community of clinician, engineer, biologist and industry thought leaders to meet the critical needs of yesterday through interdisciplinary and international cooperation and to address today's unmet clinical needs for patients with cardiovascular disease. This published Research Topic represents a curated collection of articles that uniquely attack cardiovascular disease from multiple directions, and uniformly embrace a reaffirmed interdisciplinary mission to understand and treat cardiovascular disease through regenerative medicine.

## On the Basic Science Front: Cardiovascular Disease Mechanisms and Model Systems

In abdominal aortic aneurysm (AAA), an inflammatory process drives medial degeneration, whereas in the ascending thoracic aorta, non-inflammatory smooth muscle cell loss accompanies accumulation of proteoglycans amidst elastin fragmentation (1). Martufi et al. authored a case report detailing the intra-patient heterogeneity in simulated wall stress and histopathological features of an unruptured aneurysmal specimen of human aorta from a single patient. Using finite element analysis and computational fluid dynamic simulations, mechanical wall stress, and shear stress field were estimated from computed tomographic angiography captured 2 weeks prior to elective aortic replacement. Tensile strength testing and histological analyses of resected aortic specimens from multiple regions were compared with predicted stresses. Using geometric values (e.g., wall thickness and thrombus thickness), the constitutive model exhibited good predicative value of resected specimen tensile testing. Post-operative assessment revealed appreciative regional variability in both mechanical testing and histological evaluation of elastin and collagen content and organization. The study findings are consistent with an emerging contemporary opinion that aortic size alone is an insufficient predictor of rupture risk (2, 3) and vessel heterogeneity at the tissue level in association with local biological factors could better inform on wall vulnerability.

The epi-center of human aneurysmal disease of the aorta has traditionally been the aortic media where the histopathologic hallmarks of aneurysm in both the thoracic and abdominal aorta include elastin degradation. Though the adventitia is known to provide a majority of the biomechanical support to the vessel (4), and local cell populations influence medial biology (5), there is much less known about the role of the adventitia in aneurysmal disease. Furthermore, there resides within the adventitia, several population of progenitor-like cells associated with a microvascular network known as the vasa vasorum (6) that penetrate the outer two-thirds of the medial layer in vessels of at least 29 layers of elastic lamellae (7). Focusing on specimens of human aorta, Billaud et al. made detailed morphometric analyses of the adventitial vasa vasorum and revealed several features of microvascular remodeling including reduced density of vessel, thicker walls, and increased lumen area. In accordance with these microvascular abnormalities in the adventitia, evidence of hypoxia was noted in the aortic media by detection of increased protein expression of glucose transporter 1. In a finding consistent with prior work from the same group of decreased angiogenesis-related factors in the adventitial ECM of aneurysmal human aorta (8), decrease of pro-angiogenic gene expression was associated with increased expression of the anti-angiogenic factor thrombospondin 1 (TSP-1), supporting the notion that angiogenic signaling is deficient in aneurysmal aorta. Taken together, the data bolster a theory that vasa vasorum dysfunction leads to a malperfused aortic media in aneurysmal patients. A more complete understanding in how the adventitia biologically and biomechanically influences aortic medial homeostasis may pave the way for novel therapeutic interventions that have the potential to be not only preventative, but also less invasive than the standard of care of surgical aortic replacement.

Critical to the translation of novel therapies for valvular and vascular pathologies is the development of new clinically relevant animal models. The ovine model currently serves as the gold standard for testing tissue engineered heart valves (TEHVs). Post-implantation remodeling processes *in vivo* have been largely under-studied and constitute a gap in knowledge impeding progress in TEHV optimization. In this issue, Dekker et al. comprehensively tested a sheep-specific immunohistochemical panel and validated several antibodies that detect ECM composition, cellular phenotype, and inflammatory status in ovine tissues. Inspired by the ECM composition and cellular profile of human native aortic and pulmonary valves and working specifically with formalin-fixed paraffin-embedded sections, the immunological panel was validated using the aortic valve, spleen, and kidney from normal juvenile sheep and one juvenile sheep diagnosed with endocarditis. The authors concluded that use of the immunohistological panel to evaluate ovine models will inform the researcher of ECM remodeling, cellular composition and inflammatory processes post-implantation of TEHVs.

Indeed, an understanding of native cellular composition is key to unraveling how phenotype transitions contribute to cardiovascular pathologies. From the laboratory of Hortells et al. reviewed current knowledge in the role of phenotypic changes in cardiovascular calcification. Previously thought to be a passive process, ectopic calcification exhibit features similar to biological processes of bone formation and remodeling. Focusing on the contribution of cellular phenotype, plasticity and their ability to transition to osteoblast-like cells, this review shares evidence from studies of animal disease models and human cell culture systems. Phenotypic transitions to osteoblast-like cells can occur due to fibroblast to myofibroblast conversion, EndoMT processes, and smooth muscle cells phenotypic switching. The authors raise several provocative questions pertaining to the theory that de-differentiation of a lineage-specific cell to an immature state may permit up-regulation of genes that contribute to calcium deposition and remodeling, a common denominator among these cellular transitions in cardiovascular calcification. Because cells in areas of pathological cardiovascular calcification have exhibited the ability to produce calcifying extracellular vesicles (9), the role of cell phenotype in production of extracellular vesicles whose contents either inhibit or promote mineral imbalance will be of high importance (10).

Tissue biomechanics can also influence cellular phenotype and understanding how different forces affect cell behavior is critical to developing clinically relevant cardiovascular disease models. Castellanos et al. explored changes in the cytoskeleton of bone marrow-derived stem cells (HBMSCs) under conditions mimicking fluid-induced shear stresses relevant to heart valves. Using a microfluidic channel device, HBMSCs were exposed to pulsatile shear stress (PSS) or steady shear stress (SS). When compared with no flow controls and SS exposed cells, PSS caused an increase in the number of actin filaments, filament density, and the filament angle in HBMSCs. PSS also resulted in up-regulation of klf2, a key gene involved in valvulogenesis. These findings add to our understanding in how mechanical stress affects stem cell behavior and this knowledge could help fine tune stem cell-mediated approaches to heart valve tissue engineering.

Likewise, understanding what biomechanics are at play in native valves will help to direct tissue regeneration efforts. In the case of mitral valve disease, several mechano-sensitive genes are thought to play a role and uncovering mechanisms of regulation could offer new insight into novel targeting therapeutics. Pagnozzi et al. reviewed the current literature in native mitral valve physiology and how valvular cells interpret environmental cues and interact with one another. These cell-cell and cell-environment relationships change with developmental stage and are mediated by a variety of cell surface receptors, ion channels, and structures such as cilia. Cells are also influenced by the extracellular matrix, the glycocalyx, and cell responses can be modulated by the presence of various serotonergic drugs. The authors put forth a theory of pathway integration as a likely explanation for the interplay of multiple mechanotransducing signals. Keeping the concept of dynamic reciprocity at the forefront of translational efforts, they suggest that therapeutic restoration of mechano-sensing pathway balance might be a promising direction through manipulation of the environment, modulation of how cells experience the environment or by directly altering cell-environment communication.

## Translational Horizons: Understanding and Controlling Cell Behavior for Heart Valve Engineering and Cardiac Repair

For heart valve tissue engineering, technological advances in materials-based approaches still require pursuit of unanswered questions. An incomplete understanding in how pathological mechanisms influence the body's response to implanted material is among the biggest obstacles. Bouten et al. shares a perspective on the state of the art in materials-based approaches for heart valve tissue engineering. In highlighting small-scale clinical trials, the authors raise the important, yet unanswered questions pertaining to *in situ* tissue growth and remodeling. Materials-based approaches leverage the rapid repopulation of scaffold material by endogenous cells, thus making the strategy clinically attractive and the regulatory path less complex than those dependent on inclusion of *ex vivo* cells. However, a path to translation may be limited at least in part by an incomplete understanding in how neo-tissue responds under (patho) physiological hemodynamics. The authors identified three thematic areas of outstanding challenges. First, understanding materials-driven regeneration will require new knowledge in how endogenous cells respond to implanted material. New disease models that account for inter-patient variability and enable co-culture of multiple cell types, immune cells in particular, will be highly valuable. Secondly, smart biomaterial development and rational scaffold design should recapitulate native valve function, instruct healthy tissue remodeling, and have durability across the lifespan. Lastly, predicting tissue development and growth will benefit from sophistication of computational modeling methodologies that account for material degradation and neo-tissue formation profiles, ongoing growth and remodeling, tissue architecture and signaling. Basic science inquiry remains an ongoing and integral part of the translational process for heart valve tissue engineering. A widespread skepticism of whether heart valve tissue engineering will ever come to fruition is acknowledged. The authors propose a roadmap to successful translation- one that includes integrated byways through *in vitro* and *in silico* studies, extensive pre-clinical trials and optimization en route to randomized clinical trials and cost-effectiveness evaluations amidst current valvular replacement approaches.

For any TEHV to be truly efficacious, it must resist and withstand factors and forces driving cardiovascular calcification. For example, in the setting of calcific aortic valve disease, how does one consider the cellular contribution to disease pathophysiology when designing a TEHV therapy? Jover et al. complementarily echoes Hortells et al to describe mechanisms involved in calcific aortic valve disease. The authors also highlight the pluses and minuses of various cell sources in the context of *in vitro* development of TEHVs. They encourage scaffold design with cell behavior and tissue source close in mind. Inclusion of *ex vivo* cultured mesenchymal stem cells, endothelial progenitor cells, or induced pluripotential stem cells might prevail; however, other approaches could ignore stems cells in favor of native valvular interstitial cells, valvular endothelial cells, and other native cells types. The decision-making process of choosing a cell type and source should consider several aspects including cell-graft interactions, accessibility and scalability, tissue specificity, paracrine signaling capacity, and retention on/within scaffolding biomaterials.

Recent progress in the development of biomaterials for valve, cardiac and vascular repair have shown new promise for the treatment of associated cardiovascular disease, in particular for in *vivo* delivery of exogenous cells, as expertly reviewed elsewhere (11). Understanding how biomaterials affect cell behavior is an essential component in translating new biomaterials to patients. Fibrin microthreads have recently emerged as a potential native biomaterial substrate to support stem cell growth and preserve differentiative potential. In this issue, Hansen et al. reported use of fibrin microthreads to culture human iPSC-derived cardiomyocytes (CM). Using a digital speckle tracking algorithm, the team calculated beating frequency, average and maximum contractile strain, and angle of contraction of beating iPSC-CM on fibrin microthreads and detected changes in these parameters from 7 to 21 days of culture. Seeded cells exhibited increased beating frequency, higher calcium conduction velocities, and were positive for connexin 43 expression between cells and aligned with microthread orientation. Study findings profiled seeding conditions and demonstrate temporal control of obtaining contractile behavior of cardiomyocytes. Suture needles can be threaded with fibrin microthreads, thus a regenerative approach is envisaged, where these scaffolds might effectively serve as an *in vivo* delivery system for iPSC-CM to injured or diseased myocardium.

Whether or not cells (i.e., stem/progenitor) are required for cardiovascular repair is a hotly debated topic in the field of cardiovascular regenerative medicine. Cunnane et al. addresses this controversy in the context of developing and testing of tissue-engineered vascular grafts (TEVGs). Use of an autologous stem cell population is desirable due to reduced immunologic response. However, some patient populations (e.g., the elderly, diabetic, or immunocompromized) likely possess limited stores of stem cell populations that exhibit adequate regenerative responses. Therefore, allogeneic stem cell sources are actively under consideration, with the new challenge of identifying what stem cell-derived products or functions are necessary to elicit the desired regenerative response. Excitement for stem cell therapy approach in TEVGs might be diminished in light of realization that stem cell survival post-implantation for cardiac repair was found to be relatively short-lived and the promise of successful pre-clinical studies have not translated well in clinical trials for heart failure (12). The authors share examples from the literature demonstrating the bioactivity of stem cell-derived components (e.g., secreted factors, extracellular vesicles) in TEVG studies that could be harnessed and applied to tubular scaffolds in new cell-free strategies for vascular tissue engineering.

Delivery of ECM alone, in the absence of seeded *ex vivo* cultured cells, is another viable strategy for cardiovascular regenerative medicine. This acellular approach is attractive because ECM biomaterials exhibit little no rejection *in vivo*, and it circumvents numerous obstacles related to cell procurement, propagation and cell-biomaterial interaction. A review by Svystonyuk et al. shares early strategies in cardiac regeneration and provides the rationale for acellular bioactive ECM biomaterials as an important research thrust. The epicardium appears to be a viable niche to encourage cardiac regeneration by targeting the prevalent resident fibroblast population, which comprises 20% of the myocardium and are thought to serve as active mediators of ECM-derived signaling. Prior work from the authors demonstrated reduction of scar in basic fibroblast growth factor enhanced ECM-treated rat epicardial infarcts (13). The authors suggest that acellular bioactive ECMs offer fewer translational hurdles than cell therapy-based approaches. The authors are encouraged by knowledge that ECMs represent tunable biomaterials that contain numerous bioactive signaling molecules (14) and suggest that they might potentiate endogenous repair mechanisms in the heart and are attractive biomaterials for repair of injured or diseased myocardium and perhaps other cardiovascular tissues.

## Final Perspectives on An Era of Transplantation, Uncovering Disease Mechanisms, and Tissue Engineering

What lies beyond the horizon in the current frontier of regenerative cardiovascular medicine? While great strides have been made in understanding stem cell populations and observational studies of human disease give rise to clinically-relevant and hypothesis-driven work, substantial gaps in knowledge remain regarding what pathways should be targeted for interventional therapies. Among the largest obstacles to traversing the translational divide or so-called “valley of death” to clinical application, is the tedious and tenuous pathway of commercialization and regulatory hurdles. As modern communities of academic medicine continue to cultivate fertile ecosystems that foster interdisciplinary collaboration, facilitate international cooperation, and encourage entrepreneurship, we expect these translational barriers will diminish. Ongoing basic science and translational efforts in the development of efficacious engineered tissues and organs quite possibly could help to alleviate the exceedingly high demand for donor replacement organs or ultimately eliminate the need altogether. A goal should also be to bring these future therapies to developed and non-developed regions worldwide Hutcheson et al. Given the tremendous advancements since Dr. Christiaan Barnard made the first incision in the first successful heart transplantation, the next half century should reap the fruits of the advancements in the prior 50 years.

## Author Contributions

JP drafted the editorial in consultation with JH and EA. JH, and EA edited the editorial.

### Conflict of Interest Statement

The authors declare that the research was conducted in the absence of any commercial or financial relationships that could be construed as a potential conflict of interest.
